# 3D-Volume Rendering of the Pelvis with Emphasis on Paraurethral Structures Based on MRI Scans and Comparisons between 3D Slicer and OsiriX®

**DOI:** 10.1007/s10916-020-01695-3

**Published:** 2021-01-20

**Authors:** C. M. Durnea, S. Siddiqi, D. Nazarian, G. Munneke, P. M. Sedgwick, S. K. Doumouchtsis

**Affiliations:** 1 Epsom & St Helier University Hospitals NHS Trust, Epsom, UK; 2 Luton and Dunstable University Hospitals NHS Foundation Trust, Luton, UK; 3 St George’s, University of London, London, UK; 4 Wexham Park Hospital, Slough, UK; 5 Frimley Health NHS Foundation Trust, Frimley, UK; 6 Laboratory of Experimental Surgery and Surgical Research N S Christeas, University of Athens, Medical School, Athens, Greece; 7 American University of the Caribbean, School of Medicine, Pembroke Pines, FL USA

**Keywords:** 3D slicer, OsiriX, 3D volume rendering, MRI, Paraurethral structures, Bladder

## Abstract

The feasibility of rendering three dimensional (3D) pelvic models of vaginal, urethral and paraurethral lesions from 2D MRI has been demonstrated previously. To quantitatively compare 3D models using two different image processing applications: 3D Slicer and OsiriX. Secondary analysis and processing of five MRI scan based image sets from female patients aged 29–43 years old with vaginal or paraurethral lesions. Cross sectional image sets were used to create 3D models of the pelvic structures with 3D Slicer and OsiriX image processing applications. The linear dimensions of the models created using the two different methods were compared using Bland-Altman plots. The comparisons demonstrated good agreement between measurements from the two applications. The two data sets obtained from different image processing methods demonstrated good agreement. Both 3D Slicer and OsiriX can be used interchangeably and produce almost similar results. The clinical role of this investigation modality remains to be further evaluated.

## Introduction

Pelvic floor disorders (PFD) can be associated with bothersome symptoms and affect women’s quality of life. Imaging may help in diagnosis and management [1]. Paraurethral and vaginal lesions or structures within this area have not been extensively radiologically evaluated, and may pose clinical challenges [2].

Such vaginal lesions can be associated with para-urethral Skene duct cysts [3], Müllerian cysts - congenital remnants of the paramesonephric duct [4], Gartner cysts - congenital remnants of the mesonephric duct or epidermal inclusion cysts occurring in the site of trauma or surgery [2]. Urethral diverticula usually occur on the posterior wall of the mid-urethra with a prevalence up to 6% of women [3].

Urethral, para-urethral and vaginal lesions have historically proved to be a diagnostic challenge presenting with non-specific symptoms [2]. Physical examinations are often inconclusive, so imaging is important in the diagnostic process [5].

MRI is a useful imaging modality for urethral diverticula, however differentiating a hyperintense lesion in the soft tissue surrounding the urethra as a urethral diverticulum, rather than a para-urethral or vaginal cyst can prove to be difficult [2]. A 3D volume rendered from a 2D MRI image could improve our diagnostic ability.

It has been previously reported on the feasibility of rendering 3D models of the bladder, urethra, para-urethral space and vagina [6]. In the present study we aimed to quantitatively compare 3D models created using two different image processing applications: 3D Slicer and OsiriX. We quantitatively compared linear dimensional measurements collected from 3D pelvic models of the same MRI based cross sectional images using both methods, in order to evaluate these applications and specifically to determine if in practice they can be used interchangeably.

## Materials and methods

This is a secondary analysis based on five cross sectional MRI images previously analysed, processed and published [6]. Both articles are using data from the same set of patients, however while the previous article was focussed on possibility of rendering 3D models of pelvic structures form 2D MRI cross-sectional images, this one aims to quantitatively compare 3D models created using two different image processing applications: 3D Slicer and OsiriX. This study was approved as an audit by our institutional Audit Department (Audit No 332). No consent was required to perform it.

We processed images of pelvic MRI scans from five female patients aged between 29 and 43 years old and undergoing investigations for vaginal / paraurethral masses. We initially performed a slice-by-slice examination of images in axial, sagittal, and coronal view to clarity the MRI anatomical appearance of the structures of interest. Then the 3D - appearance of each structure was related to each of 3 planes consecutively to confirm the finding again. Inversion recovery (IR), T1 and T2-weighted spin-echo images were obtained from a 1.5 Tesla magnet (Signa HDxt, GE Medical Systems, Milwaukee WI, USA). All 5 patients positioned with feet supine first underwent MRI sequences including IR, T1 and T2-weighted axial, coronal and sagittal; high resolution T2-weighted coronal and axial and T1 fat-saturated axial fast spin echo sequences. Every set contained approximately 20 images with slice thicknesses of between 3 and 5 mm and 4–5.5 mm spacing between slices. The data used were in Digital Imaging and Communication in Medicine (DICOM) format.

Two open source image processing applications were used for image analysis, visualisation, segmentation, label mapping and 3D volume rendering. The first one was 3D Slicer v.3.4.0 (Brigham and Women’s Hospital, Harvard Medical School, Boston, MA, USA). The second one was OsiriX (Pixmeo, Bernex, Switzerland).

The detailed technique of cross-sectional anatomy identification, volume rendering and creation of 3D models was described previously.

The primary objective was to quantitatively compare 3D models using two different image processing applications 3D Slicer and OsiriX using the Bland-Altman method [7].

### Linear measurements

These image processing applications allow to record linear measurements of structures of interest on 2D MRI scans as well as on 3D volume rendered models. Measurements were recorded to two decimal places. For enhanced accuracy, fiducial markers were placed within the 3D model and each dimension was measured in the sagittal, coronal and axial plane, and an average value was calculated.In the same way, the distance between the distal aspect of the mass and the external urethral meatus and the distance between the proximal aspect of the para-urethral pathology and the bladder neck were calculated. All measurements were entered onto a Microsoft ® Excel (2010) database for further analysis.

### Quantitative data analysis

The agreement between the two software measurements was analysed using the Bland-Altman method [7]. Measurements were also compared with findings from the original 2D MRI and clinical reports.

## Results

**Fig. 1:**
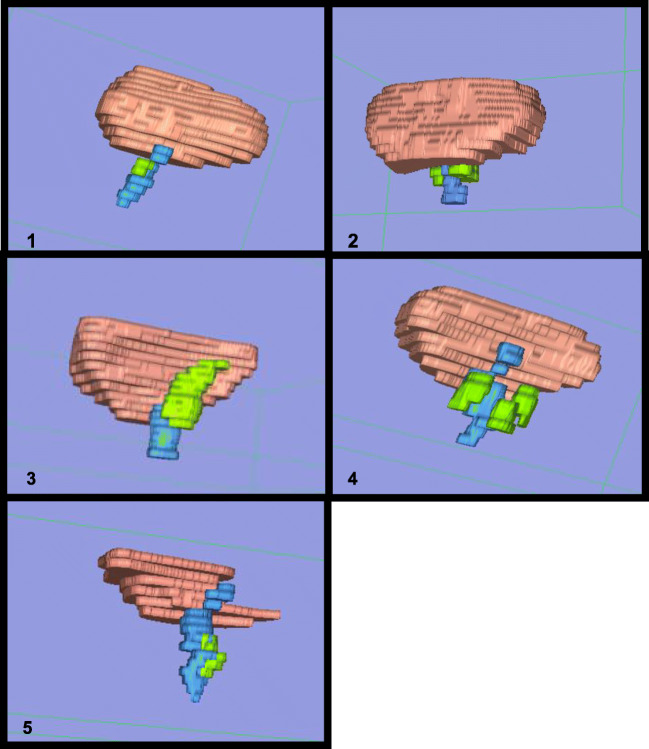
3-Dimension Volume rendering in OsiriX. (Bladder (pink), urethra (blue), lesion – (green), detailed below.). Patient 1 – urethral diverticulum. Patient 2–2 urethral bulking and a sub-urethral cyst. Patient 3 – Gartner cysts. Patient 4 – three para-urethral cysts. Patient 5 – urethral diverticulum

Patient demographic data and clinical information is demonstrated in Table 1. Five 3D models were successfully rendered using OsiriX and 3D Slicer (Fig. 1). These represented clinical cases of two urethral diverticula, urethral bulking with a sub-urethral cyst, Gartner cysts, and a case of three para-urethral cysts. The obtained models clearly demonstrated the size of the lesions and their anatomical relationships with the bladder and urethra. The mean dimensional measurements obtained from 3D Slicer and OsiriX are presented in Table 2.

**Table 1:** Demographic data and clinical information

Serial NO	Demographics	Clinical presentation	Clinical diagnosis	MRI report	Surgical Report	Histopathology
1	43 yrs., Black, Para 3	Mixed urinary incontinence, Palpable sub-urethral mass	Urethral Diverticulum 6 mm × 9 mm	Urethral Defect at 5 o’clock Small collapsed diverticulum 6x9mm	Urethral diverticulum 10x20mm at 5 o’clock, 1 cm from external meatus	20x10x10mm urethral diverticulum
2	43 yrs., White European, Para 3	Recurrent stress urinary incontinence Sub-urethral mass	Grade 2 urethrocele, ? sub-urethral cyst	3 septated cysts around proximal urethra, 14 × 13, 15 × 10 and 13x9mm Likely bulking material	Not applicable	Not applicable
3	39 yrs. White European, Para 2	Stress urinary incontinence, Vaginal mass	3 cm cyst in R anterior fornix	R lateral vaginal wall cyst 3.8 × 1.5 × 1.4 cm with septations	4-5 cm Right anterolateral vaginal wall cyst	Not applicable
4	39 yrs., Chinese, Para 2	Vaginal lump, Minor stress urinary incontinence	1x Grade 1 cystocele 2x para-urethral cysts: 1x Right sub-urethral 0.5x1cm 1x Right lateral wall 0.5 × 1cm	3x para-urethral cysts No urethral connection Right sided: 15 mm, 11 mm Left sided: 12 mm	EUA*: 2 para-urethral cysts, no connection to urethra Not excised	Not applicable
5	29 yrs., White European, Para 1	Vaginal lump, Intermittently draining milky fluid via urethra.	3-4 cm urethral diverticulum	Urethral diverticulum 8x23mm	Urethral diverticulum 3-4 cm	25x20mm urethral diverticulum

*EUA = examination under anesthesia

**Table 2:** The mean dimensional measurements (mm) from 3D Slicer and OsiriX

Structure	Height				Width				Depth				Distance from prox. Lesion to bladder neck				Distance from distal lesion to external meatus			
	3D-^*^ Slicer	OsiriX*	Diffe-rence^§^	Ave-rage^§^	3D Slicer	OsiriX	Difference	Ave-rage	3D Slicer	OsiriX	Difference	Ave-age	3D Slicer	OsiriX	Difference	Ave-rage	3D Slicer	OsiriX	Difference	Ave-rage
Patient 1																				
Bladder mean measurements	63.3	63.15	0.15	63.23	85.7	94.25	−8.55	89.98	73.65	75.4	−1.75	74.53								
Urethra mean measurements	36.2	36.8	−0.6	36.5	16.1	13.7	2.4	14.9	16.25	14.15	2.1	15.2								
Lesion mean measurements	6.46	6.45	0.01	6.45	9.05	8.91	0.15	8.98	5.2	4.56	0.65	4.88	19.1	18.2	0.9	18.65	21.5	21.7	−0.2	21.6
Patient 2																				
Bladder mean measurements	57.75	57.7	0.05	57.73	91.15	95.3	−4.15	93.23	59.25	60.4	−1.15	59.83								
Urethra mean measurements	58.15	53.95	4.2	56.05	13.9	14.1	−0.2	14	11.34	9.96	1.38	10.65								
Lesion left mean measurements	16.75	15.7	1.05	16.23	11.1	10.39	0.72	10.74	10.5	10.25	0.25	10.38	5.23	6.83	−1.6	6.03	33.9	32.8	1.1	33.35
Lesion right mean measurements	14.35	14.15	0.2	14.25	8.7	8.58	0.13	8.64	8.22	8.63	−0.41	8.43	9.38	9.63	−0.25	9.51	33	30.3	2.7	31.65
Lesion rear mean measurements	15.1	14.45	0.65	14.78	16.6	15.35	1.25	15.98	14.8	14.05	0.75	14.43	14.5	17	−2.5	15.75	35.3	36.3	−1	35.8
Patient 3																				
Bladder mean measurements	44	43.2	0.8	43.6	84.25	85.75	−1.5	85	46.4	48.15	−1.75	47.28								
Urethra mean measurements	34.05	30.05	4	32.05	13.7	15.3	−1.6	14.5	13	11	2	12								
Lesion mean measurements	38.3	37.6	0.7	37.95	20.2	19.45	0.75	19.83	14.25	13.7	0.55	13.98	36.7	35.5	1.2	36.1	30.9	30.2	0.7	30.55
Patient 4																				
Bladder mean measurements	38.18	36.65	1.53	37.42	71.16	67.7	3.46	69.43	36.55	43.7	−7.15	40.13								
Urethra mean measurements	35.15	34.45	0.7	34.8	15.8	15.5	0.3	15.65	13.68	13.25	0.43	13.47								
Lesion left mean measurements	14.66	15.35	−0.69	15.01	8.06	7.6	0.47	7.83	11.96	11	0.96	11.48	21.4	21.6	−0.2	21.5	21.6	17.5	4.1	19.55
Lesion right mean measurements	13.96	13.1	0.86	13.53	9.34	8.74	0.6	9.04	11.74	12.15	−0.41	11.95	20.9	22	−1.1	21.45	22.1	24.6	−2.5	23.35
Lesion inferior mean measurements	11.26	10.41	0.85	10.84	10.02	9.27	0.75	9.65	9.63	7.46	2.17	8.55	24.2	25.5	−1.3	24.85	18	15.7	2.3	16.85
Patient 5																				
Bladder mean measurements	12.68	13.4	−0.72	13.04	48.7	53.5	−4.8	51.1	43.31	44.9	−1.59	44.11								
Urethra mean measurements	36.56	33.55	3.01	35.06	15.28	15.2	0.08	15.24	13.96	13.95	0.01	13.96								
Lesion mean measurements	21.09	17.9	3.19	19.5	11.11	9.18	1.94	10.14	8.36	7.7	0.66	8.03	18.7	17.6	1.1	18.15	5.12	7.1	−1.98	6.11

*- The mean dimensional measurements (mm) of the bladder, urethra and lesion in each patients as recorded from 3D Slicer and OsiriX

^§^ - The difference and average between these measurements was calculated to two decimal places

On initial analysis we plotted the results from OsiriX against those from 3D Slicer on a regression line The Osirix measurements had good agreement to the ones obtained from 3D Slicer based on the Bland-Altman method. Analysis included the mean of each dimension and the graphs produced can be seen in Figs. 2.1–2.11. Visually all data points were clustered around the line of equality with apparent minimal difference.

**Fig. 2:**
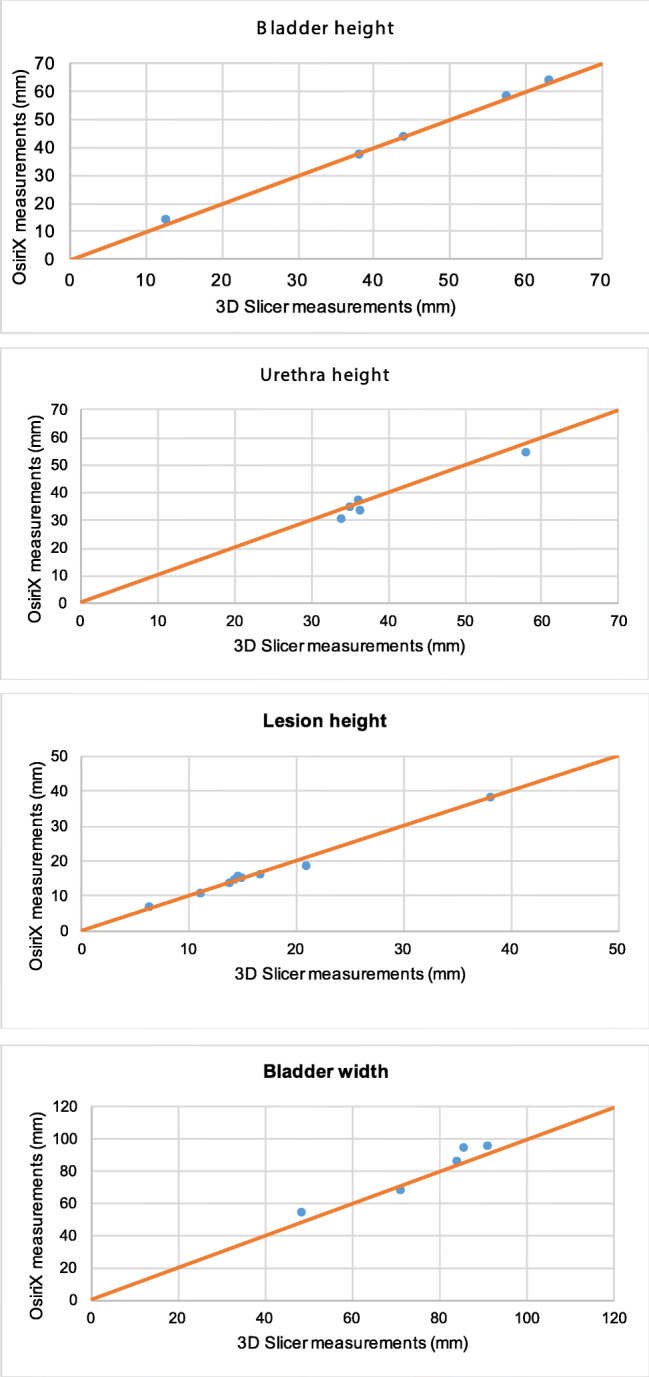

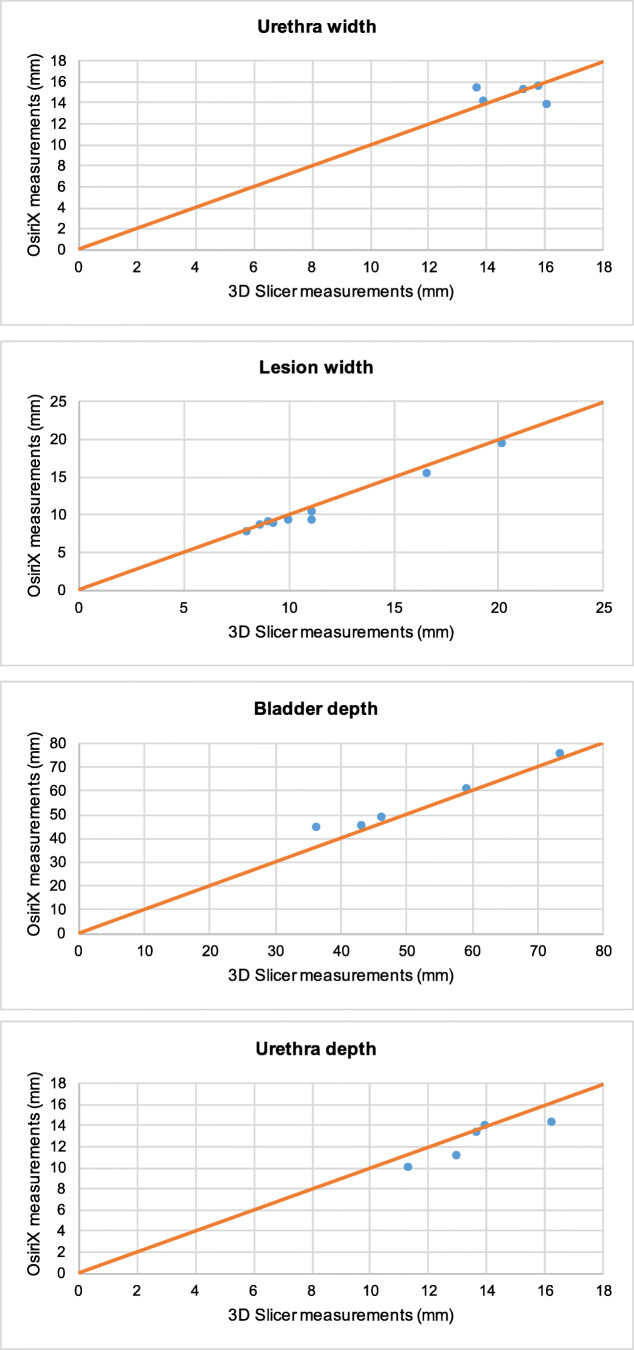

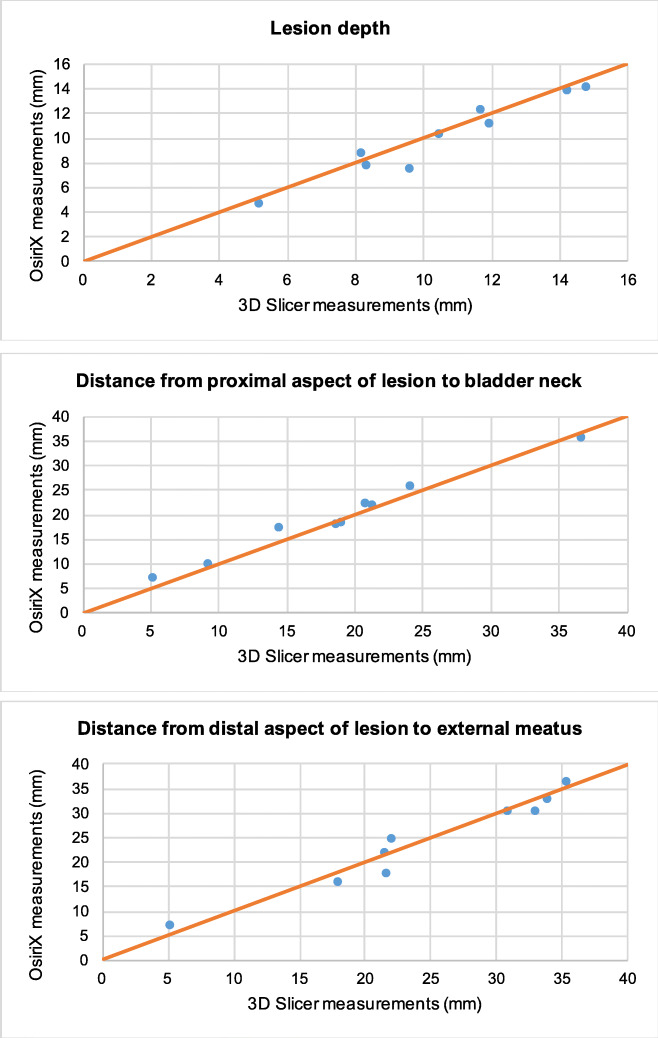
OsiriX and 3D Slicer Mean Dimensional Measurements in each patient. **2.1**: In each of the 5 patients, the mean bladder height measurement in 3D Slicer plotted against the mean bladder height measurement in OsiriX (mm). **2.2**: In each of the 5 patients, the mean urethra height measurement in 3D Slicer plotted against the mean urethra height measurement in OsiriX (mm). **2.3**: In each of the 5 patients, the mean lesion height measurement in 3D Slicer plotted against the mean lesion height measurement in OsiriX (mm). **2.4**: In each of the 5 patients, the mean bladder width measurement in 3D Slicer plotted against the mean bladder width measurement in OsiriX (mm). **2.5**: In each of the 5 patients, the mean urethra width measurement in 3D Slicer plotted against the mean urethra width measurement in OsiriX (mm). **2.6**: In each of the 5 patients, the mean lesion width measurement in 3D Slicer plotted against mean lesion width measurement in OsiriX (mm). **2.7**: In each of the 5 patients, the mean bladder depth measurement in 3D Slicer plotted against the mean bladder depth measurement in OsiriX (mm). **2.8**: In each of the 5 patients, the mean urethra depth measurement in 3D Slicer plotted against the mean urethra depth measurement in OsiriX (mm). **2.9**: In each of the 5 patients, the mean lesion depth measurement in 3D Slicer plotted against the mean lesion depth measurement in OsiriX (mm) **2.10**: In each of the 5 patients, the mean distance from the most proximal part of the lesion to the bladder neck in measurements from 3D Slicers plotted against the mean distance from the most proximal part of the lesion to the bladder neck in measurements from OsiriX (mm). **2.11**: In each of the 5 patients, the mean distance from the most distal aspect of the lesion to the external urethral meatus in measurements from 3D Slicers plotted against the mean distance from the most distal aspect of the lesion to the external urethral meatus in OsiriX (mm)

**Fig. 3:**
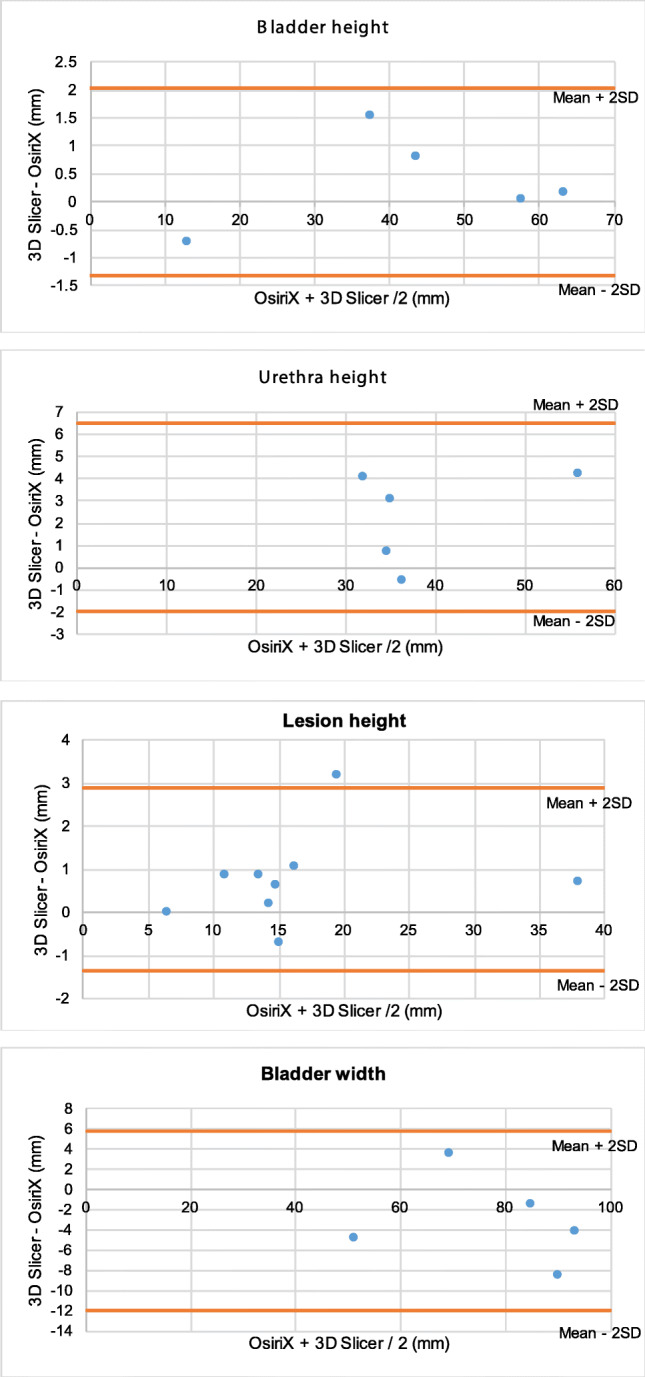

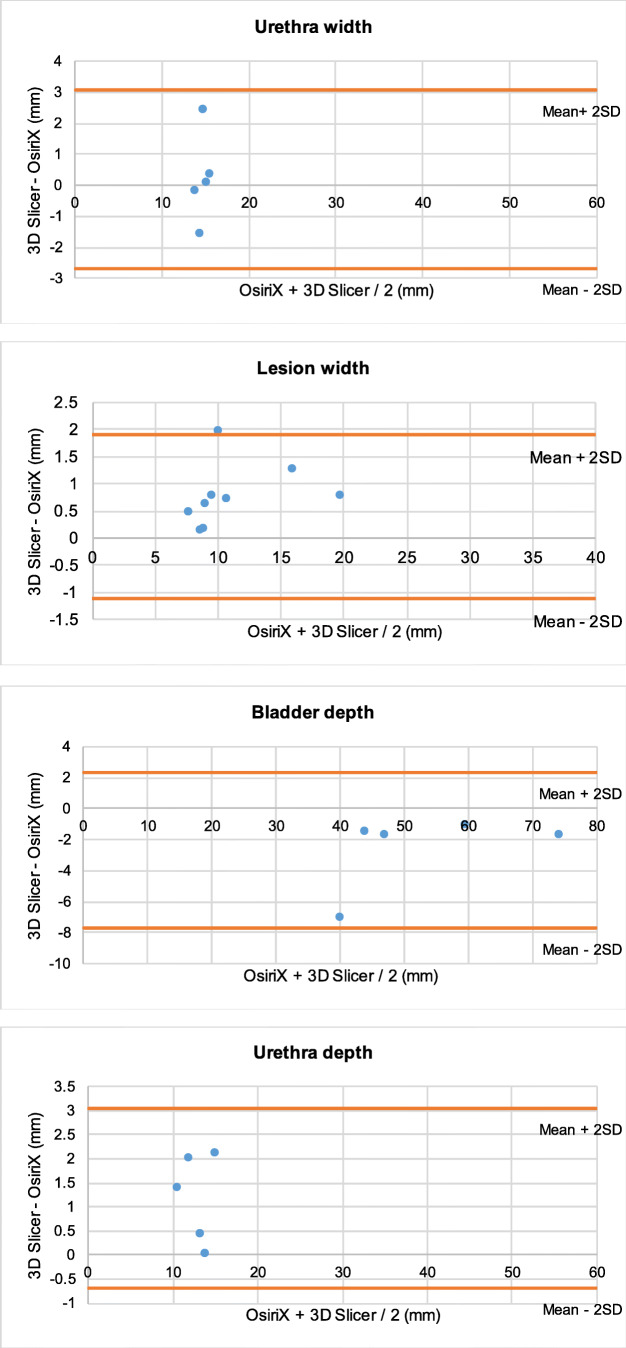

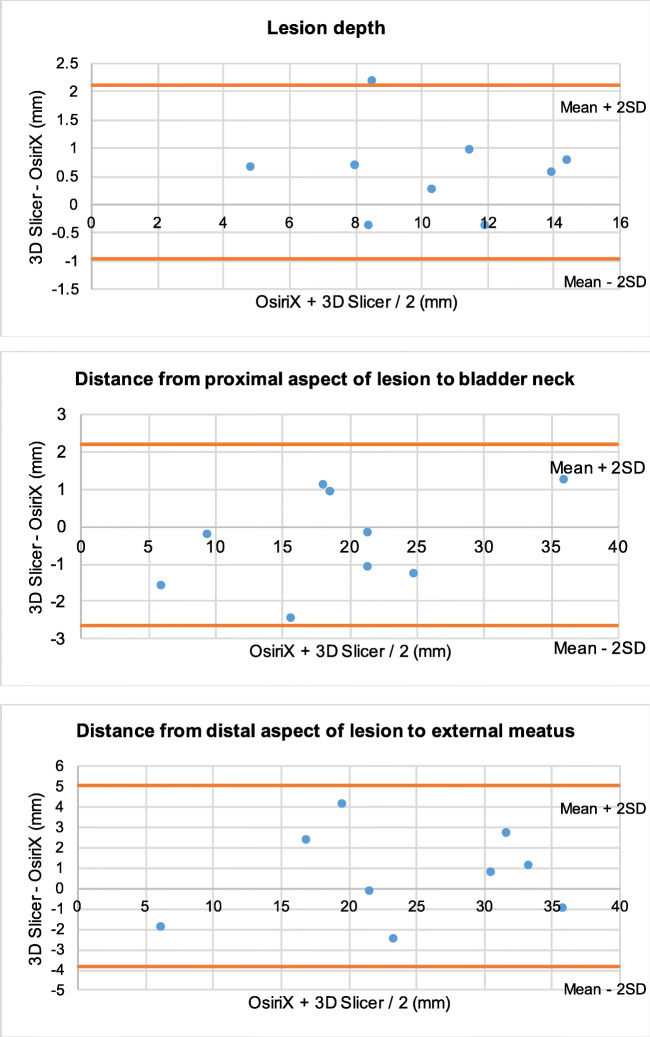
Bland-Altman Plots - mean of each dimension as recorded in OsiriX and 3D Slicer against the difference between mean measurements in the two software programs **3.1**: In each of the 5 patients, the average of the mean bladder height measurements from 3D Slicer and OsiriX, plotted against the difference between the mean bladder height measurements in 3D Slicer and OsiriX (mm). **3.2**: In each of the 5 patients, the average of the mean urethra height measurements from 3D Slicer and OsiriX, plotted against the difference between the mean urethra height measurements in 3D Slicer and OsiriX (mm). **3.3**: In each of the 5 patients, the average of the mean lesion height measurements from 3D Slicer and OsiriX, plotted against the difference between the mean lesion height measurements in 3D Slicer and OsiriX (mm). **3.4**: In each of the 5 patients, the average of the mean bladder width measurements from 3D Slicer and OsiriX, plotted against the difference between the mean bladder width in 3D Slicer and OsiriX (mm). **3.5**: In each of the 5 patients, the average of the mean urethra width measurements from 3D Slicer and OsiriX, plotted against the difference between the mean urethra width in 3D Slicer and OsiriX (mm). **3.6**: In each of the 5 patients, the average of the mean lesion width measurements from 3D Slicer and OsiriX, plotted against the difference between the mean lesion width in 3D Slicer and OsiriX (mm). **3.7**: In each of the 5 patients, the average of the mean bladder depth measurements from 3D Slicer and OsiriX, plotted against the difference between the mean bladder depth in 3D Slicer and OsiriX (mm). **3.8**: In each of the 5 patients, the average of the mean urethra depth measurements from 3D Slicer and OsiriX, plotted against the difference between the mean urethra depth in 3D Slicer and OsiriX (mm). **3.9**: In each of the 5 patients, the average of the mean lesion depth measurements from 3D Slicer and OsiriX, plotted against the difference between the mean lesion depth in 3D Slicer and OsiriX (mm). **3.10**: In each of the 5 patients, the average of the mean distance from the most proximal aspect of the lesions to the bladder neck from 3D Slicer and OsiriX, was plotted against the difference between the mean distance from the most proximal aspect of the lesions to the bladder neck in 3D Slicer and OsiriX (mm). **3.11**: In each of the 5 patients, the average of the mean distance from the most distal aspect of the lesions to the external meatus from 3D Slicer and OsiriX, was plotted against the difference between the mean distance from the most distal aspect of the lesions to the external meatus in 3D Slicer and OsiriX (mm)

The agreement between the data obtained from the two programs was assessed using the Bland-Altman method. They are presented in appendix B. Only three data points were lying outside of 2 standard deviations of the mean (Fig. 3).

## Discussion

The main aim of this study was to assess the extent of agreement between the measurements obtained from rendered 3D volume structures using the image processing applications OsiriX and 3D Slicer, to determine if they can be used interchangeably in practice. Data analysis demonstrated a high degree of agreement between the measurements from both methods. The Bland-Altman plots illustrated that the majority of data points clustered demonstrating no difference between measurements, although measurements from both methods were not completely identical. The analysis demonstrated that none of the methods is superior to the other.

As mentioned previously, urethral and para-urethral lesions are often difficult to diagnose and differentiate on clinical examination [8]. Creation of virtual spatial imaging permits construction of high resolution 3D models‚ which can be reviewed in detail to maximise interpretation and diagnostic value of the images. These methods may allow a detailed view of pathology present in multiple planes whilst still appreciating the distance of the lesions to pelvic landmarks and organs. Besides pre-surgical planning this technology may also play a role in patient counselling and medical education.

Three dimensional volume rendering (3DVR) of cross-sectional two dimensional scans visibly demonstrates the spatial relationship between different anatomic structures. It also allows accurate volumetric measurement [9]. It has simplified and improved interpretation of complex radiological images in a way accessible to clinicians. This is a revolutionary leap in medical imaging technologies [10]. Imaging becomes more complex nowadays, being multidimensional (CT and MRI) and multi-modal (e.g. PET-CT). This challenges conventional PACS (picture archiving and communication system) viewer. PACS was a digital replacement of film-based medical images [11]. However its use is increasingly restricted to small series of 2D scans. It is time consuming and not cost-effective reviewing a large amount of images. There was a need for image processing applications making them suitable for a quick and efficient analysis. High-tech 3D work stations have been available recently, but their access is restricted to specialised radiologists. The development of user friendly, accessible and free image processing applications such as OsiriX and 3D Slicer may improve the way clinicians can interpret images. It may assist diagnosis and management of relevant pelvic floor disorders and anatomical lesions. Comprehensive medical training is not required to create these models, so it is accessible to a wide range of health care professionals with online tutorials guides being available.

However, OsiriX is only compatible with Macintosh operating systems, while 3D Slicer is available across all platforms. Considering possible future clinical implementation of this technique of image analysis, it is important that users have access to high quality software and image viewer and analysis tools regardless of the operating system. Both methods can generate nearly similar results, which is encouraging for widespread use of this technology.

Due to the small sample size, this study is best considered as exploratory rather than a confirmatory one. Therefore, the findings should be interpreted with caution and further research is required. Although 3D imaging techniques are currently available (ultrasound and MRI), performing and interpreting such modalities, requires additional resources, equipment and training which may not be widely and readily available. The image processing applications evaluated and assessed for their feasibility in this particular clinical context, offer the option of using two dimensional cross sectional MRI images for the assessment of spatial anatomical relationships and measurements, which would be otherwise not possible to undertake with the two dimensional cross sectional images.

## Strengths and limitations

The 3D measurement, allows greater accuracy in determination of the position, extent and relationship of the mass to adjacent anatomical structures. The measurements obtained from a conventional 2D image cannot be as accurate for description of spatial structure because diagonal distances running between the slices cannot be measured.

In a scan with a slice thickness of 3-5 mm and interslice distance of 4–5.5 mm, there is a high margin of error for any potential method. Extrapolating that distance is likely to be too large to be acceptable in a clinical or pre-surgical setting.

The models rendered from OsiriX and 3D Slicer were generated by different operators, therefore, there may be a degree of operator bias during the manual segmentation phase. In this study however, this was minimised by consulting the same radiologist to validate that the manual segmentation was performed correctly. This improved accuracy between the measurements recorded between data sets. In a wider application of 3DVR, operator bias in skilled and unskilled professionals may pose a significant limitation to the technique.

The sample size of this study was small and accordingly anatomical variation between patients with PFD could not be accurately assessed. A larger sample could investigate the anatomical differences in the pelvic floor anatomy in different population groups.

Between slices of MRI scans there was a 3-5 mm gap. This rendered volumetric images with ‘step-like’ external appearance rather than being smooth, decreasing the visual precision of the model. In the models generated from 3D Slicer ‘smoothing’ compensated for this, however in OsiriX no such tool is available. However slice gaps of 2 mm can improve accuracy [12].

The time and resources taken to create the models must be taken into consideration. Although it is not an excessively labour intensive process, the appropriate software and expertise is required to successfully create the models. The 2D MRI scans used in this study were limited to 32 slices per series. If thinner slices were taken, this would require a far more time intensive process.

This study was based on 5 scans only‚ being by design an exploratory one rather than a confirmatory. Studies with much larger number of subjects and measurements may be required to confirm these findings. This would be a recommendation for a future study design.

## Conclusions

There was a high degree of agreement between the resulting two data sets obtained from different image processing applications. All statistical tests demonstrated minimal differences between the measurements undertaken using both applications. The Bland-Altman method for assessing agreement demonstrated minimal differences between the methods undertaken using the applications of 3D Slicer and OsiriX. These methods can be used interchangeably and produce almost similar results.

## Data Availability

Yes
